# Determination of Spatiotemporal Gait Parameters Using a Smartphone’s IMU in the Pocket: Threshold-Based and Deep Learning Approaches

**DOI:** 10.3390/s25144395

**Published:** 2025-07-14

**Authors:** Seunghee Lee, Changeon Park, Eunho Ha, Jiseon Hong, Sung Hoon Kim, Youngho Kim

**Affiliations:** 1 Department of Biomedical Engineering, Yonsei University, Wonju 26493, Republic of Korea; fhrm502@yonsei.ac.kr (S.L.); cepark915@yonsei.ac.kr (C.P.); 2 Division of Data Science, Yonsei University, Wonju 26493, Republic of Korea; statha@yonsei.ac.kr; 3 Department of Medicine, Wonju College of Medicine, Yonsei University, Wonju 26426, Republic of Korea; g7284@naver.com (J.H.); kimrehab@yonsei.ac.kr (S.H.K.)

**Keywords:** gait event, spatiotemporal parameter, IMU, deep learning, knowledge distillation, hemiplegic stroke

## Abstract

This study proposes a hybrid approach combining threshold-based algorithm and deep learning to detect four major gait events—initial contact (IC), toe-off (TO), opposite initial contact (OIC), and opposite toe-off (OTO)—using only a smartphone’s built-in inertial sensor placed in the user’s pocket. The algorithm enables estimation of spatiotemporal gait parameters such as cadence, stride length, loading response (LR), pre-swing (PSw), single limb support (SLS), double limb support (DLS), and swing phase and symmetry. Gait data were collected from 20 healthy individuals and 13 hemiparetic stroke patients. To reduce sensitivity to sensor orientation and suppress noise, sum vector magnitude (SVM) features were extracted and filtered using a second-order Butterworth low-pass filter at 3 Hz. A deep learning model was further compressed using knowledge distillation, reducing model size by 96% while preserving accuracy. The proposed method achieved error rates in event detection below 2% of the gait cycle for healthy gait and a maximum of 4.4% for patient gait in event detection, with corresponding parameter estimation errors also within 4%. These results demonstrated the feasibility of accurate and real-time gait monitoring using a smartphone. In addition, statistical analysis of gait parameters such as symmetry and DLS revealed significant differences between the normal and patient groups. While this study is not intended to provide or guide rehabilitation treatment, it offers a practical means to regularly monitor patients’ gait status and observe gait recovery trends over time.

## 1. Introduction

Walking is one of the most common human physical activities, and gait monitoring has proven highly beneficial in various fields such as healthcare and sports [1]. Regular walking reduces the risk of chronic diseases like cardiovascular conditions and diabetes [2], strengthens muscles, and improves balance and flexibility. Furthermore, walking enhances nervous system functions and helps prevent neurodegenerative diseases such as dementia [3]. For elderly individuals, maintaining gait abilities is essential for daily life, as gait disorders increase the risk of falls. Gait events can be divided into four main events: initial contact (IC), toe off (TO), opposite initial contact (OIC), and opposite toe off (OTO). Spatiotemporal gait parameters include stride length, cadence, loading response (LR), single limb support (SLS), pre-swing (PSw), and swing phase and symmetry [4]. LR corresponds to the first double limb support period, while PSw represents the second double limb support (DLS) period. Stride length refers to the distance traveled during one gait cycle, measured from one IC to the next IC. Cadence, the number of steps per minute, can be calculated based on the duration of one gait cycle. Double limb support represents the period when both feet are in contact with the ground during walking. The first double limb support, also known as LR, occurs from IC to OTO, and the second double limb support, also known as PSw, occurs from OIC to TO. In normal walking, LR constitutes approximately 10% of the total gait cycle. SLS time refers to the period when only one foot is in contact with the ground, occurring between OTO and OIC. It corresponds to approximately 40% of the gait cycle and matches the swing phase of the opposite leg. Gait symmetry was calculated as the ratio of IC to OIC within a gait cycle, with symmetric gait typically showing a ratio of approximately 50% [5]. This calculation is formally presented in Equation (1). Stance phase refers to the duration from IC to TO, during which the foot remains in contact with the ground, comprising approximately 62% of the gait cycle. The swing phase, the period from TO to the next IC, accounts for about 38% of the cycle. Monitoring these spatiotemporal gait parameters is crucial for the early diagnosis of various diseases [6]. For instance, changes in stride length and double support time can indicate aging and fall risks [7,8]. Abrupt decreases in walking speed may also be associated with cardiovascular diseases and other age-related conditions [9].

(1) $$\;Gait\;symmety\;(\%)=\frac{\left|\;IC\;(\%)-OIC\;(\%)\;\right|}{One\;gait\;cycle\;(\%)}$$

Gait analysis traditionally requires expensive equipment, such as 3D motion analysis systems and force plates, installed in specialized facilities [10]. Wearable sensors such as IMU sensors have emerged as an effective alternative due to their ease of use and ability to be worn comfortably on the body. In particular, smartphones are passively carried in everyday life, enabling natural and continuous data collection in real-world environments. For example, Kim et al. [11] proposed an algorithm to estimate gait parameters using insoles embedded with inertial sensors. They detected IC and TO for each foot, reporting a maximum error of 3 ms. Data were collected at a sampling rate of 100 Hz across three treadmill speeds. To analyze gait regularity, continuous wavelet transforms (CWT) were applied, and the signal peaks were interpreted as IC and TO events. McCamley et al. [12] placed an IMU on the lower trunk and used vertical acceleration signals with continuous wavelet transforms to detect IC and TO. They reported errors of −6 ± 24 ms and −29 ± 26 ms compared to a force plate. Maqbool et al. [13] attached sensors to the calf and used angular velocity signals to detect gait events, validating them with in-shoe pressure sensors. Ledoux [14] applied a threshold-based method with IMUs mounted laterally on the calf and reported highly precise timing errors of less than 2%. Romijnders et al. [15] validated IC and TO detection using an IMU sensor attached near the shank, operating at a sampling rate of 200 Hz. They compared the results with those from reflective marker systems and reported mean detection errors of approximately 11 ms for IC and 2 ms for TO. In their follow-up study [16], they developed a deep learning-based model utilizing IMU sensors placed on the shank and ankle to detect IC and TO events for each foot. They reported a maximum average error of approximately 3 ms. By applying a window-shifting technique to the inertial sensor data collected during gait and employing a Temporal Convolutional Network (TCN), a CNN-based deep learning model, they achieved greater accuracy compared to their previous work. De Miguel-Fernández et al. [17] estimated gait events using an IMU embedded in a shank-mounted exoskeleton and compared them statistically with 3D motion capture data. However, their method required the exoskeleton to be worn on both legs and did not provide direct one-to-one comparisons of predicted versus actual gait events, making it difficult to directly compare with the present study. Strick et al. [18] proposed an algorithm to detect IC and TO using IMU sensors placed on the thigh. By analyzing hip and knee joint angles simultaneously, they identified the local minimum of the knee angle as the IC point and determined TO within a specific window where the joint angle decreased. The reported mean errors were 4.1 ms for IC and 1.4 ms for TO. Voisard et al. [19] designed an algorithm to detect gait events, including IC and TO, using an IMU sensor mounted on the dorsum of the foot. They employed Dynamic Time Warping (DTW) to align gait patterns, reporting errors below 10 ms for healthy participants and 20–30 ms for patients. Gurchiek et al. [20] mounted sensors on the thigh and identified gait events based on acceleration thresholds; their method required sensors on both legs. These studies primarily focused on detecting IC and TO, but accurate estimation of full spatiotemporal parameters like SLS or PSw requires additional events such as OIC and OTO. However, relatively few studies have attempted to detect all four gait events using smartphone sensors in real-world settings, leaving a practical gap in comprehensive and accessible gait monitoring. Yang et al. [5], for instance, detected all four gait events using a KAFO-embedded IMU and validated the method with a motion capture system. In contrast, our study used a smartphone placed in the user’s pocket. This study proposes a hybrid method that combines threshold-based rules with SVM-based feature fusion, enabling robust detection of all four gait events with reduced computational complexity.

To simplify gait event detection and parameter calculation, various studies have explored smartphone-based approaches. Silsupadol et al. [21] utilized smartphone accelerometer data to detect IC and compute spatiotemporal gait parameters, including step time, step length, cadence, and symmetry. However, these studies fixed smartphones to bags or waistbands and only detected IC and TO, limiting their practical applications. Research has shown that the most common location where users store their smartphones is in their pockets [22,23]. Ward et al. [22] investigated smartphone storage habits and found that 91 out of 174 participants (52%) kept their smartphones in their pockets. Similarly, Redmayne’s survey [23] reported that more than half of users carried their smartphones in pockets below the waist. Pepa et al. [1] validated algorithms from previous studies [12,24,25] using smartphone-embedded inertial sensors placed in pockets or on the waist. They detected IC with errors ranging from −7 ms to 12 ms and calculated stride time and length with errors of −2 ms to 0 ms and 1.13 cm to 0.48 cm, respectively.

This study aims to detect all four key gait events using smartphone IMU sensors placed in the pocket and to calculate spatiotemporal gait parameters such as stride length, cadence, LR, PSw, SLS, DLS, and stance phase and symmetry. Unlike previous methods that rely on dedicated sensors and fixed positions, this approach leverages a device already carried by users in daily life. The main contributions of this study are as follows: (1) comprehensive gait event detection using a smartphone IMU at a low sampling rate; (2) validation in both healthy individuals and patients; and (3) a hybrid model combining threshold-based and deep learning techniques. These results highlight the potential for real-world and non-intrusive gait monitoring in clinical or daily environments. Although this study does not aim to evaluate the outcomes of rehabilitation programs directly, it provides a framework for monitoring how much a patient’s gait deviates from normal patterns, which may assist in tracking gait recovery progress over time.

## 2. Materials and Methods

### 2.1. Participants

To develop a gait monitoring algorithm using smartphone-embedded inertial sensors placed in pockets, Gait experiments were conducted in 20 healthy adults who had no musculoskeletal disorders and exhibited normal gait patterns. To verify the algorithm, data from 14 hemiparetic stroke patients were also collected and analyzed. All experiments were approved by the Institutional Review Board of Yonsei University (1041849-202409-BM-187-04), and informed consent was obtained from all participants.

### 2.2. Equipment

**Figure 1:**
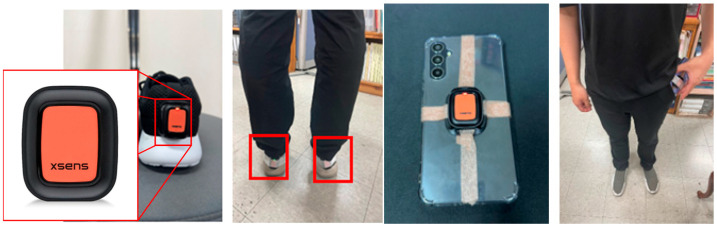
Sensor position. The red box on the left highlights the Xsens Dot attached to the shoe, and the right side shows an IMU sensor and a smartphone placed inside a pocket.

Inertial sensor data were collected from a smartphone placed in the pocket using a custom-developed Gait Param application, which recorded accelerometer and gyroscope data at a sampling rate of 50 Hz. The application was developed using the Flutter platform and is designed to measure data from the smartphone’s built-in IMU sensors. It is compatible with both Android and iOS devices. The smartphones used were Galaxy A34 (SM-A346N, Samsung Electronics Co., Ltd., Suwon, Republic of Korea). To validate gait events and parameters, commercial IMU sensors (Xsens DOT, Movella Holdings Inc., Henderson, NV, USA) were attached to the heels of both shoes and the back of the smartphone, recording data at 60 Hz. The data were resampled to 50 Hz and synchronized with the smartphone data. Figure 1 illustrates the placement of the commercial IMU sensors.

### 2.3. Experimental Methods

#### 2.3.1. Normal Gait

Normal gait data were collected from 20 healthy participants (height: 170.8 ± 7.0 cm, weight: 68.4 ± 14.2 kg, age: 23.8 ± 2.0 years). Participants walked on a treadmill (H/P/Cosmos-Air Walk system, H/P/Cosmos sports and medical GmbH, Nussdorf-Traunstein, Germany) at three different speeds (3, 4, 5 km/h) for 1 min each, and repeated 5 times. Before data collection, participants practiced walking at these speeds to become familiar with the treadmill.

#### 2.3.2. Patient Gait

Experiments were conducted with 13 hemiparetic stroke outpatients walking approximately 9 m in a straight line. Walking on flat ground was chosen instead of treadmill walking to avoid the risk of falls in patients. Table 1 presents patient information, including Functional Ambulation Classification (FAC) levels ranging from 0 to 5. Level 0 indicates no independent walking, while levels 4 and 5 indicate independent indoor and outdoor walking. One patient (P01) with an FAC level of 1 was excluded from the analysis.

### 2.4. Gait Event and Gait Parameter Detection Algorithms

#### 2.4.1. Reference Gait Events

**Figure 2:**
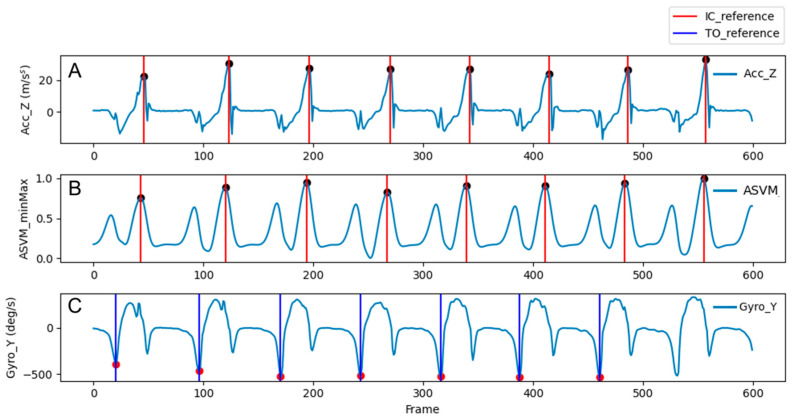
Examples of reference gait event determination: (**A**) shows the anterior-posterior acceleration signal and reference IC points, (**B**) shows the ASVM signal and reference IC points, and (**C**) shows the medial-lateral angular velocity signal and reference TO points.

The reference gait events were extracted to validate the algorithm using inertial sensors attached to each heel. IC was determined using the maximum value of the anterior-posterior (*Z*-axis) acceleration signal [26,27,28]. Figure 2A shows the *Z*-axis acceleration signal and the extracted reference IC points, while Figure 2B represents the signal of the acceleration sum vector magnitude (ASVM). As observed in Figure 2A,B, IC points align with the maximum peaks in both the *Z*-axis acceleration and ASVM signals. In cases of patient gait, IC may not always occur at the heel. To address this, IC was determined using ASVM signals, which represent the impact better. The second-order Butterworth low-pass filter with a cutoff frequency of 3 Hz was applied to reduce sensor noise [29]. TO was identified using the minimum value of the medial-lateral (*Y*-axis) angular velocity signal [28,30,31]. Figure 2C illustrates an example showing the reference TO points. The angular velocity signal, maintained close to zero during the stance phase, exhibits a sudden change just before TO, and reaches a minimum at TO. Following the swing phase, the signal reached a maximum near IC and returns close to zero during the stance phase.

The sum vector magnitude (SVM) was selected as a feature vector to minimize the impact of smartphone movements inside the pocket. The ASVM and angular velocity SVM (GSVM) were computed using Equations (2) and (3), respectively. A second-order Butterworth low-pass filter (3 Hz) was applied to reduce potential noise [29].

(2) $$ASVM=\sqrt{{{Acc}_{X}}^{2}+{{Acc}_{Y}}^{2}+{{Acc}_{Z}}^{2}}$$

(3) $$GSVM=\sqrt{{{Gyro}_{X}}^{2}+{{Gyro}_{Y}}^{2}+{{Gyro}_{Z}}^{2}}$$

where ${Acc}_{X}$, ${Acc}_{Y}$, and ${Acc}_{Z}$ represent the acceleration values along the X, Y, and Z axes, and ${Gyro}_{X}$, ${Gyro}_{Y}$, and ${Gyro}_{Z}$ represent the angular velocity values along the X, Y, and Z axes, respectively.

#### 2.4.2. Threshold-Based Gait Event Detection

**Figure 3:**
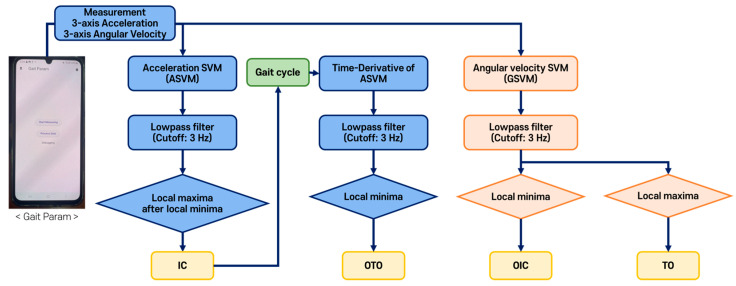
Flowchart of the threshold-based gait event detection algorithm.

Figure 3 illustrates the flowchart of the threshold-based algorithm. It detected four gait events through a series of processes after measuring the three-axis accelerations and three-axis angular velocities from the smartphone IMU sensor placed in the pocket.

**Figure 4:**
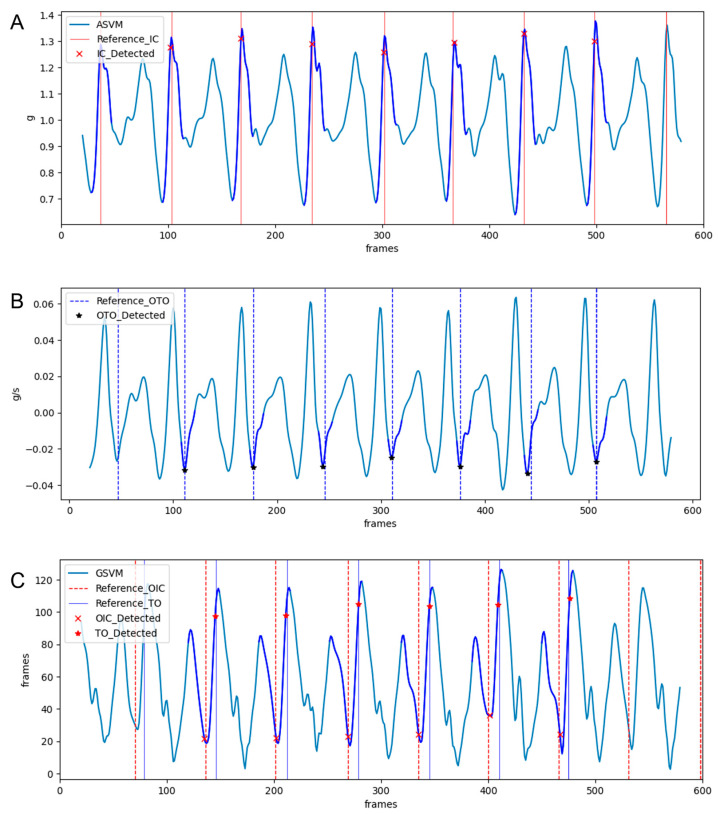
Examples of threshold-based gait event detection: (**A**) shows the ASVM graph and IC detection points, (**B**) shows the ASVM derivative graph and OTO detection points, and (**C**) shows the GSVM graph and OIC and TO detection points. The blue lines in each figure indicate the temporary window used to detect the corresponding event.

Figure 4 presents graphs measured using the smartphone’s built-in inertial sensor inside the pocket, including reference event points and the points detected by the algorithm. Figure 4A shows the ASVM graph alongside the reference and the detected points of IC. Similar maximum peaks were observed near the reference points of TO, while the maximum peaks in ASVM occur near the reference points of IC. The algorithm detected the minimum peaks preceding the reference points of IC to distinguish the IC and TO. Consequently, IC detection involves identifying the minimum peaks in the ASVM signal and determining the subsequent maximum peaks as IC points. The threshold for finding the minimum peaks in ASVM was set to 60% of the difference between the minimum value across the entire ASVM signal and its maximum value. The sections marked in dark blue on the graph indicate the regions where both minimum and maximum peaks were detected. If the difference between the maximum and minimum peaks exceeds 50% of the total ASVM signal range, the IC point is identified. The red ‘x’ marks on the graph represent the IC points detected based on this approach. Figure 4B shows the temporal derivative of the ASVM graph used to detect OTO, along with the OTO reference and detected points. OTO is a gait event that occurs after IC and was found near the minimum peaks in the ASVM derivative values. Although two minimum peaks appear in the ASVM derivative graph, OTO is determined as the minimum peak following the IC, considering the sequential nature of gait events. Figure 4C shows the GSVM graph used to detect OIC and TO, along with the reference and detected points for these events. The reference points for OIC and TO are identified as the minimum and maximum peaks of GSVM, respectively. Since the gait events OTO, OIC, and TO occur in sequence within a single gait cycle, the last minimum and maximum peaks detected in GSVM before the next IC are designated as OIC and TO, respectively.

Four major gait events detected by the algorithm were validated by calculating the error rate with the reference events using Equation (4). The detected gait events were also used to compute spatiotemporal gait parameters such as cadence, stride length, LR, PSw, SLS, DLS, and symmetry. These parameters were compared with those calculated from the reference events to validate the algorithm.

(4) $$Error\;rate\;\left(\%\right)=\frac{Detected\;frame-Reference\;frame}{Number\;of\;freames\;in\;a\;gait\;cycle}\times 100\;$$

where the detected frame refers to the frame of the detected gait event, and the reference frame refers to the frame of the reference event. The denominator represents the total number of frames in a gait cycle. A frame refers to an IMU data measured at a frequency of 50 Hz.

A paired *t*-test was conducted between the reference and the detected events to statistically analyze the algorithm’s performance.

#### 2.4.3. DL-Based Gait Event Detection

The dataset used for the DL-based model consisted of 1345 samples, each representing a single gait cycle. Among them, 1125 samples were used for training and 220 samples were used for testing.

**Figure 5:**
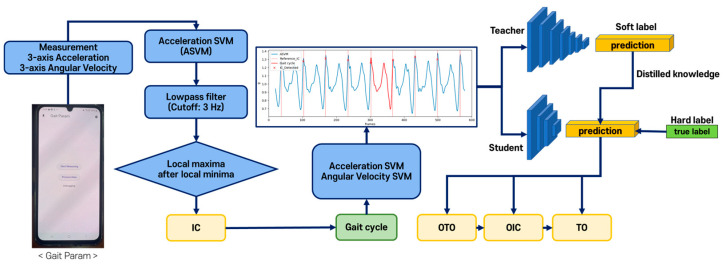
Flowchart of DL-based gait event detection algorithm.

Figure 5 illustrates the overall process of the DL-based gait event detection algorithm. IC was first detected using the threshold-based method to extract the gait cycle. The remaining events are then predicted using the DL model, and spatiotemporal gait parameters are computed using the detected gait events. Among the 20 healthy participants, a 4:1 ratio was used to divide the participants into training and test groups. For the patient group, excluding one participant, 8 out of the remaining 12 were included in the training set, and 4 were assigned to the test set. Data augmentation techniques such as noise addition and data duplication were applied to the patient gait samples, since the number of patient gait samples was smaller than that of the normal samples. Gaussian noise with a mean of 0 and a variance of 0.01 was added to augment the data, following the method described by Iglesias et al. [32]. The error rate calculation for algorithm validation follows Equation (3), and the comparison of gait parameters follows the same method as the threshold-based approach.

Knowledge distillation (KD) was applied to the DL model for lightweighting [33]. KD involves transferring knowledge from a large teacher model to a smaller student model, maintaining compactness while enhancing performance. The DL models consisted of CNN (convolutional neural network) and LSTM (long short-term memory) architectures. The CNN model, first proposed by LeCun et al. [34], is an architecture widely used in image-related fields and is highly effective for extracting features from data. While 2-D CNNs are typically used in image processing, 1-D CNNs have recently gained popularity for time-series data analysis [35]. LSTM, an architecture designed to address the vanishing gradient problem in RNNs (Recurrent Neural Networks) for time-series data processing, is commonly used in applications such as time-series analysis and natural language processing [36].

The teacher model comprised four 1-D convolutional layers, three LSTM layers, and two dense layers. The convolutional layers contained 32, 64, 128, and 64 filters, in that order, with a stride of 3. Leaky ReLU was used as the activation function, and Max Pooling was applied between convolutional layers to prevent overfitting. Each LSTM layer contained 64 units, and Global Average Pooling was applied between the LSTM and dense layers. The final dense layer used a Softmax activation function to predict the probabilities of gait events. Categorical Cross-Entropy was applied as the loss function for training. The student model consisted of two 1-D convolutional layers and one dense layer. The convolutional layers contained 8 and 16 filters, in that order, with the same stride of 3. The student model also used the Softmax activation function, and Categorical Cross-Entropy was applied. Both the teacher and student models were trained with a learning rate of 0.001, a batch size of 32, and the Adam optimizer. The algorithm implementation and neural network training were conducted using TensorFlow in Python. The computer used for analysis was equipped with an Intel (Santa Clara, CA, USA) i7-12700 processor (2.1 GHz), 32 GB RAM, an NVIDIA (Santa Clara, CA, USA) GeForce RTX 3060 GPU, and ran on Windows 11.

The threshold-based algorithm detected IC and extracted each gait cycle, which was then used as input data for the DL model. The gait cycle data were normalized to a size of 100, and only two feature vectors, ASVM and GSVM, were used to minimize axis information. Thus, all input data were standardized to a size of 100 × 2.

The DL model utilized a single extracted gait cycle as input data, allowing the model to learn consistent patterns in gait rather than employing a shifting-window technique. Multi-task learning was implemented to train and predict the timings of individual gait events (OTO, OIC, and TO), enabling the model to independently learn patterns for each event. Through this approach, a single input sample produces three different outputs corresponding to each event type using a single model [37]. Additionally, the model utilized the SoftMax activation function in the output layer to predict the probabilities of gait events across the entire gait cycle.

**Figure 6:**
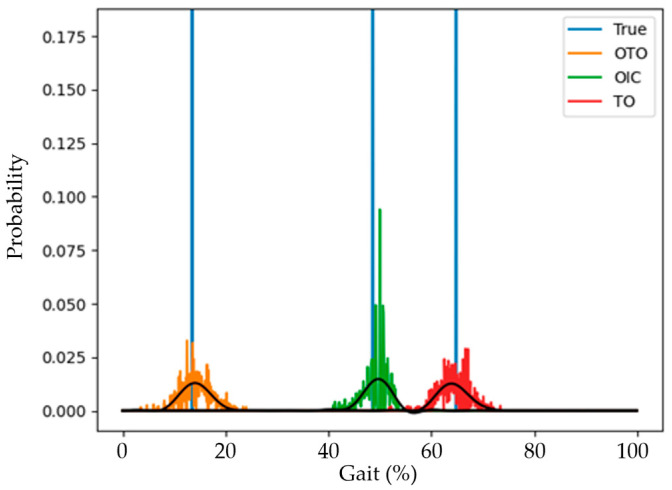
Example of DL-based model output.

Figure 6 represents an example of the predicted probabilities and reference event timings from the model’s output layer. The light blue vertical lines represent the reference gait event timings, while the orange, green, and red bars represent the probabilities of OTO, OIC, and TO, respectively. The black line indicates the trendline of each graph, with the point of highest probability used to determine the detected timing. The *y*-axis represents the probability of each timing, and the *x*-axis represents the gait cycle.

#### 2.4.4. Statistical Analysis

This study focused on detecting gait events and parameters and validating their accuracy through the algorithms operated on both normal and patient walks. Therefore, we collected the reference data and the detected data for four gait events in three different ways from normal people and hemiplegic patients. We performed a paired *t*-test on each person’s paired data to control for the heterogeneity of walking by variables such as age, gender, patient condition, etc. Furthermore, we also obtained the corresponding confidence intervals. In addition, a two-sample *t*-test was performed to statistically compare the gait data between normal and patient groups, allowing for an objective assessment of gait differences across populations. All statistical analyses in this study were conducted using the scipy.stats library in Python 3.9.13.

## 3. Results

The teacher model contained a total of 566,808 parameters (6783 KB), whereas the KD model was smaller, with 51,456 parameters (231 KB).

Each gait cycle was normalized to 100%, with the first IC set at 0% and the next IC at 100%. Table 2 presents the gait event error rates, the results of paired t-tests comparing the reference and detected timings, and the corresponding 95% confidence intervals. In Table 2, each value is presented as “error rate (mean ± standard deviation), *p*-value, and [95% confidence interval]”. For normal gait, the threshold-based algorithm showed minimal errors (e.g., TO: 0.3%, OTO: −0.1%), while the DL-based teacher model showed similar or better performance. The KD model demonstrated slightly larger deviations, particularly for TO (1.9%). In patient gait, error rates were generally higher, especially in the DL-based models, where OIC and TO showed differences of up to −3.9% and −3.5%, respectively.

Key spatiotemporal gait parameters (cadence, stride length, LR, PSw, SLS, DLS, and symmetry) are summarized in Table 3 and Table 4. Cadence and stride length showed nearly identical values between reference and detected data; thus, only the threshold-based results are shown. Other parameters showed small deviations, with the DL-based teacher model generally closer to the reference values than the KD model. The † symbol in Table 4 indicates statistically significant differences (*p*-value < 0.05) between the normal and patient groups. Cadence and stride length were excluded from the statistical analysis because walking speed conditions differed between the two groups—treadmill for normal participants and overground for patients. The two-sample *t*-test results for gait parameters confirmed statistically significant differences in all gait parameters, demonstrating that the spatiotemporal gait parameters differ between healthy individuals and patients. The patient group exhibited a longer DLS duration compared to the normal group and showed lower gait symmetry.

## 4. Discussion

In this study, four essential gait events were detected using the built-in inertial sensor of a smartphone placed in a pocket, and essential spatiotemporal gait parameters were determined. Two approaches were used for gait event detection: a threshold-based method and a DL-based method. Both approaches extracted SVM features to minimize dependence on the sensor orientation and applied a low-pass filter with a cutoff frequency of 3 Hz to eliminate noise effects.

Compared to other gait events, IC was detected with the highest consistency, whereas OTO and OIC exhibited greater variation in timing accuracy. This discrepancy may result from the less distinctive signal characteristics associated with contralateral foot events. These findings highlight an inherent challenge in detecting OTO and OIC, which has been largely overlooked in previous studies.

In the statistical analysis of gait event detection, the threshold-based method showed statistically significant differences for most gait events except OIC in normal walking, while in patient walking, no significant differences were observed except for IC. The teacher model exhibited non-significant differences for OTO, as well as TO in normal gait, but only OTO remained non-significant in the patient gait. The KD model demonstrated significant differences for all events except OTO in both normal and patient gait. These results suggest that the teacher model maintained relatively higher accuracy and generalization across different gait patterns compared to the threshold-based and KD models. The teacher model showed an error rate within 1% for normal gait, while the maximum error in patient gait occurred at OIC by approximately 4%. This outcome can be attributed to the larger proportion of normal gait data in the training set compared to patient gait data, as well as the greater variability in patient gait patterns, which resulted in lower training weights [38]. For normal gait, the KD model revealed the maximum error of 1.9% at TO, whereas the largest error of −3.2% occurred at OIC for patient gait.

The two-sample *t*-test results between the normal and patient groups show that the DLS duration in the patient group is longer than that of healthy individuals, and their gait symmetry is lower. Although patients with FAC levels of 4 or higher were capable of independent walking, noticeable asymmetry in their gait led to statistically distinct values from those of healthy individuals. In particular, a significant difference in DLS and gait symmetry further indicated structural differences in gait patterns between the two groups. As DLS requires the detection of all four major gait events, it offers a more detailed assessment of gait quality than symmetry alone, making it a valuable parameter for clinical gait monitoring.

For normal gait parameters, cadence and stride length showed a very small difference between the reference values and the detected values, with an error of approximately 1%. The cadence in normal gait was higher than in patient gait, whereas the stride length was shorter. This result might be due to the fact that the normal gait was measured on a treadmill, while the patient’s gait was measured on level ground, leading to a tendency for stride length to be shorter at the same cadence on a treadmill [39].

The threshold-based algorithm revealed the maximum difference of 0.8% in gait parameters such as LR, PSw, SLS, DLS, and symmetry for both normal and patient gait data compared to the reference values, demonstrating its excellent performance. However, the accuracy of the algorithm may decrease when significant acceleration or angular velocity occurs during movements other than walking, as the thresholds are set based on the relative maximum and minimum values of the signals.

For normal gait, the DL-based teacher model resulted in less than 1% error of gait parameters, while the KD model showed a maximum error of 2.1% for PSw. In general, patients have large deviations in gait parameters. The DL-based teacher model resulted in a maximum of 4.0% error for symmetry, while the KD model showed a maximum error of approximately 3.3%. The performance in symmetry was slightly lower, since data from patients with gait symmetry close to 50% were also included in the training, and the number of patient data with reduced symmetry was relatively small. The KD model was learned from the teacher model’s information and showed slightly larger error rates than the teacher model, but reduced the model size successfully by about 96%, achieving model compression.

Among the 12 remaining patients, 10 were classified as level 5 on the FAC, indicating that they were capable of independent walking, while the other 3 were level 4, able to walk independently only on flat surfaces. TO timings of hemiplegic patients with independent walking were found to be similar to those of healthy individuals, but the OIC occurred earlier compared to healthy individuals, indicating reductions in gait symmetry. The patient data used for training and validating the models were from nine patients, and only cases with a relatively large amount of measurement data were included.

Table 5 summarizes prior studies on gait event detection using IMU sensors, focusing on their sensor placement, sampling rate, and detection accuracy. Most of the earlier works primarily targeted ipsilateral events such as IC and TO, and required trunk-, shank-, or thigh-mounted sensors with high sampling rates to achieve acceptable accuracy. These setups, while effective, often relied on controlled environments and body-fixed sensor locations, limiting their everyday usability.

Yang et al. [5] is one of the few studies that detected all four gait events using a KAFO-embedded IMU. In contrast, the present study achieved competitive results using only a smartphone placed in the user’s side pocket, with no fixed attachment or dedicated wearable. Despite using a lower sampling rate of 50 Hz, our study successfully detected all four gait events with error rates comparable to or better than prior studies, demonstrating both practical applicability and robustness in unconstrained, real-world settings.

This study has several limitations. First, walking tasks were conducted under different conditions: a treadmill for healthy participants and flat overground for patients. This discrepancy may influence gait characteristics and should be considered when interpreting the results [40]. Second, the current approach focuses on smartphone placement in the side pocket. However, studies have shown that many users also carry their smartphones in handbags, which may affect sensor orientation and data quality. Additionally, if the smartphone is placed in locations other than the side pocket—such as a jacket pocket or the back pocket of pants—it may produce different signals, making it difficult for the system to operate reliably. Further research is needed to validate the method’s reliability in various storage positions. Finally, this study does not provide direct clinical rehabilitation guidance or personalized intervention. Instead, it serves as a practical and accessible tool to monitor how closely a patient’s gait resembles that of healthy individuals, potentially supporting progress tracking in real-world contexts.

## 5. Conclusions

This study proposes a hybrid approach that combines threshold-based rules and deep learning to detect four key gait events (IC, OTO, OIC, and TO) and estimate spatiotemporal gait parameters (LR, SLS, PSw, DLS, and symmetry) using a smartphone’s built-in IMU sensor. By integrating traditional threshold-based methods with artificial intelligence, the proposed approach enhances gait event detection accuracy. Even a lightweight KD model achieved a high event detection accuracy with a maximum error rate below 1.9% for the normal group. The method demonstrated reliable performance even at a low sampling rate of 50 Hz. In addition, statistical analysis confirmed significant differences in spatiotemporal gait parameters such as DLS and symmetry between the normal and patient groups. Notably, the proposed approach exhibited high performance with a maximum error rate below 3.9% not only in normal gait but also in patient gait. Through this study, we demonstrate that the proposed method enables accurate assessment of the four major gait events and spatiotemporal gait parameters using a smartphone kept in the pocket, without being affected by sensor orientation. Therefore, it can be conveniently used to evaluate gait status in both healthy individuals and patients in real time.

## Figures and Tables

**Table 1:** Patient information.

Patient No.	Height (cm)	Weight (kg)	Age (Years)	Sex	Type of Stroke	Onset (Months)	Side	FAC (Level)
P01	165	71	83	M	Infarct	14	Right	1
P02	161	73	65	F	Infarct	45	Left	5
P03	170	80	58	M	Infarct	106	Right	5
P04	175	70	53	M	Infarct	32	Right	5
P05	167	75	56	M	Infarct	38	Right	5
P06	164	78	68	M	Infarct	8	Right	5
P07	173	75	41	M	Hemorrhage	236	Left	5
P08	169	70	77	M	Hemorrhage	260	Right	4
P09	162	62	67	M	Infarct	67	Right	4
P10	163	65	66	M	Infarct	167	Left	4
P11	164	68	54	F	Hemorrhage	141	Left	5
P12	156	48	35	F	Hemorrhage	287	Right	5
P13	158	59	51	F	Infarct	116	Left	5
Average	165.1	69.1	60.7	-				
SD	5.2	8.2	13.1					

**Table 2:** Error rates (%), *p*-value, and 95% confidence intervals of detecting gait events.

Subjects	Algorithm		IC	OTO	OIC	TO
Normal	Threshold-based		−0.3 ± 3.7% ** *p* < 0.01 [0.5, 2.0]	−0.1 ± 4.4% ** *p* < 0.01 [1.3, 4.8]	0.4 ± 4.5% 0.09 [−0.1, 1.7]	0.3 ± 4.3% ** *p* < 0.01 [1.0, 3.8]
	DL-based	Teacher	-	−0.2 ± 3.6% 0.56 [−0.7, 0.4]	0.5 ± 3.1% * 0.04 [0.0, 0.9]	−0.2 ± 4.3% 0.52 [−0.8, 0.4]
		KD		0.7 ± 2.7% ** *p* < 0.01 [0.3, 1.1]	−0.3 ± 2.4% 0.16 [−0.61, 0.1]	1.9 ± 2.9% ** *p*< 0.01 [1.5, 2.3]
Patient	Threshold-based		0.7 ± 3.6% * 0.02 [−1.0, −0.1]	−1.1 ± 5.9% 0.20 [−0.4, 1.8]	−0.4 ± 5.8% 0.32 [−0.64, 2.0]	−0.3 ± 6.4% 0.91 [−1.5, 1.3]
	DL-based	Teacher	-	−1.4 ± 6.8% 0.24 [−3.9, 1.0]	−3.9 ± 4.6% ** *p* < 0.01 [−5.6, −2.3]	−3.5 ± 5.2% ** *p* < 0.01 [−5.4, −1.6]
		KD		−0.8 ± 5.7% 0.44 [−2.8, 1.3]	−3.2 ± 5.6% ** *p* < 0.01 [−5.2, −1.2]	−2.6 ± 5.7% * 0.01 [−4.7, −0.6]

* indicates a statistical difference between reference and detected gait events (*p* < 0.05). ** indicates a statistical difference between reference and detected gait events (*p* < 0.01).

**Table 3:** Results of gait parameters: cadence and stride length.

Threshold-Based		Gait Parameters	
		Cadence (steps/min)	Stride Length (cm)
Normal	Reference	106.6 ± 11.4	126.8 ± 18.5
	Detected	107.7 ± 11.9	125.6 ± 18.3
Patient	Reference	96.9 ± 14.1	134.2 ± 22.5
	Detected	96.9 ± 14.7	134.4 ± 22.6

**Table 4:** Results of gait parameters: LR, PSw, SLS, DLS and symmetry.

	Model		LR (%)	PSw (%)	SLS (%)	DLS (%)	Symmetry (%)
Normal	Threshold -based	Reference	10.7 ± 3.3	12.8 ± 3.2	38.1 ± 2.4	23.5 ± 4.4	48.8 ± 2.4
		Detected	10.5 ± 3.3	13.1 ± 3.3	38.0 ± 3.9 *	23.6 ± 4.8	48.6 ± 3.2
	DL-based	Reference	13.4 ± 1.8	17.0 ± 2.7	35.7 ± 3.1	30.5 ± 3.5	49.2 ± 2.5
		Teacher	13.6 ± 2.9	17.7 ± 4.3	35.1 ± 4.1	31.2 ± 5.4	48.7 ± 3.0
		KD	12.8 ± 2.0	14.9 ± 1.3	36.6 ± 2.0	28.2 ± 2.9	49.4 ± 1.0
Patient	Threshold -based	Reference	17.0 ± 4.2 †	17.0 ± 3.8 †	28.8 ± 5.4 †	34.0 ± 6.5 †	45.8 ± 4.4 †
		Detected	17.2 ± 3.1 †	17.5 ± 5.3 †	28.0 ± 8.0 †	34.2 ± 7.0 †	45.2 ± 8.1 †
	DL-based	Reference	17.7 ± 5.1 †	19.9 ± 3.0 †	27.1 ± 4.0 †	37.5 ± 5.4 †	44.7 ± 4.8 †
		Teacher	19.1 ± 4.6 †	19.4 ± 3.7 †	29.6 ± 4.3 †	40.2 ± 5.9 †	48.7 ± 3.1 †
		KD	18.5 ± 3.2 †	19.3 ± 5.9 †	29.5 ± 3.5 †	40.7 ± 5.9 †	48.0 ± 2.3 †

* indicates a statistical difference between reference and detected parameters (*p* < 0.05). † represents a statistical difference between the normal and patient groups (*p* < 0.05).

**Table 5:** Previous studies on detection of gait events.

		[12]	[15]	[13]	[14]	[20]	[5]	This Study	
								Threshold	Teacher
Sensor position		Trunk	Shank	Shank	Shank	Thigh	Thigh	Pocket	
Sampling rate (Hz)		100	200	100	500	128	60	50	
IC	Error rate (%)	2.0			−1.7 ± 0.6		−0.1 ± 0.6	−0.3 ± 3.7	
	Error time (ms)	6 ± 24	−11 ± 57	11 ± 18		39 ± 28	−1 ± 8	−8.3 ± 67	
TO	Error rate (%)	3.0			−1.8 ± 0.6		−0.2 ± 1.9	0.3 ± 4.3	−0.2 ± 4.3
	Error time (ms)	−29 ± 26	2 ± 31	−8 ± 35		28 ± 28	−3 ± 24	9.9 ± 70.7	−0.4 ± 5.4
OIC	Error rate (%)						1.0 ± 1.3	0.4 ± 4.5	0.2 ± 2.7
OTO	Error rate (%)						−1.1 ± 2.9	−0.1 ± 4.4	0.7 ± 3.0

## Data Availability

The data presented in this study are available upon request from the corresponding author. The data are not publicly available because the authors are continuing the study.
